# Supplementary figures and images for: Identification and validation of apoptosis-related genes in acute myocardial infarction based on integrated bioinformatics methods
Source: PeerJ. 2024 Dec 4;12:e18591. doi: 10.7717/peerj.18591 (PMC11624842; doi:10.7717/peerj.18591)

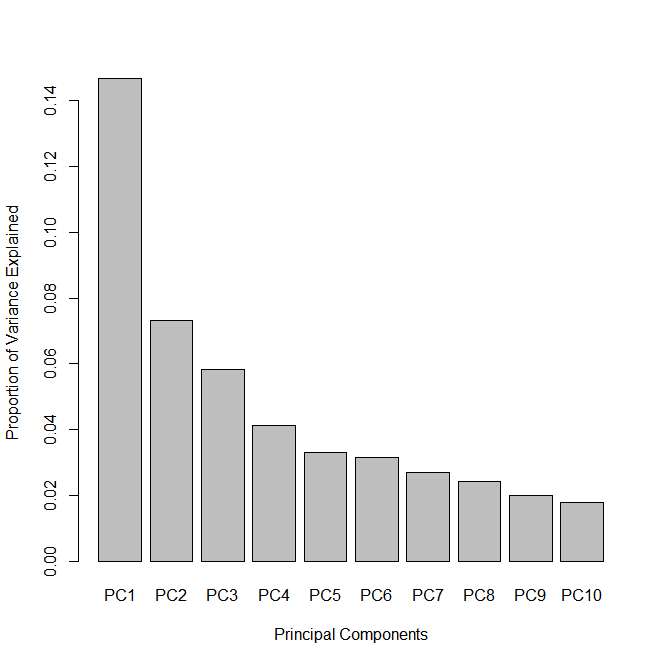

Supplement: Supplemental Information 3 [file peerj-12-18591-s003.docx]
