# Supplementary material for: Identification and validation of apoptosis-related genes in acute myocardial infarction based on integrated bioinformatics methods
Source: PeerJ. 2024 Dec 4;12:e18591. doi: 10.7717/peerj.18591 (PMC11624842; doi:10.7717/peerj.18591)
Supplement: Supplemental Information 6 [file peerj-12-18591-s006.pdf]

ITEM TO CHECK	IMPORTANCE	CHECKLIST
**EXPERIMENTAL DESIGN**		
Definition of experimental and control groups	**E**	Line 249
Number within each group	**E**	Line 249
Assay carried out by core lab or investigator's lab?	D	
Acknowledgement of authors' contributions	D	
**SAMPLE**		
Description	**E**	
Volume/mass of sample processed	D	
Microdissection or macrodissection	**E**	Line 256
Processing procedure	**E**	
If frozen - how and how quickly?	**E**	Line 256
If fixed - with what, how quickly?	**E**	
Sample storage conditions and duration (especially for FFPE samples)	**E**	Line 256
**NUCLEIC ACID EXTRACTION**		
Procedure and/or instrumentation	**E**	
Name of kit and details of any modifications	**E**	Line 274
Source of additional reagents used	D	
Details of DNase or RNAse treatment	**E**	Line 275
Contamination assessment (DNA or RNA)	**E**	Line 275
Nucleic acid quantification	**E**	Line 275
Instrument and method	**E**	Line 276
Purity (A260/A280)	D	
Yield	D	
RNA integrity method/instrument	**E**	Line 276
RIN/RQI or Cq of 3' and 5' transcripts	**E**	Line 276
Electrophoresis traces	D	
Inhibition testing (Cq dilutions, spike or other)	**E**	
**REVERSE TRANSCRIPTION**		
Complete reaction conditions	**E**	
Amount of RNA and reaction volume	**E**	Line 277
Priming oligonucleotide (if using GSP) and concentration	**E**	Line 277
Reverse transcriptase and concentration	**E**	Line 277
Temperature and time	**E**	Line 277
Manufacturer of reagents and catalogue numbers	D	
Cqs with and without RT	D*	
Storage conditions of cDNA	D	
**qPCR TARGET INFORMATION**		
If multiplex, efficiency and LOD of each assay.	**E**	
Sequence accession number	**E**	
Location of amplicon	D	
Amplicon length	**E**	
In silico specificity screen (BLAST, etc)	**E**	
Pseudogenes, retropseudogenes or other homologs?	D	
Sequence alignment	D	
Secondary structure analysis of amplicon	D	
Location of each primer by exon or intron (if applicable)	**E**	
What splice variants are targeted?	**E**	
**qPCR OLIGONUCLEOTIDES**		
Primer sequences	**E**	Line 282
RTPrimerDB Identification Number	D	
Probe sequences	D**	
Location and identity of any modifications	**E**	Line 282
Manufacturer of oligonucleotides	D	
Purification method	D	
**qPCR PROTOCOL**		
Complete reaction conditions	**E**	
Reaction volume and amount of cDNA/DNA	**E**	Line 279
Primer, (probe), Mg++ and dNTP concentrations	**E**	Line 279
Polymerase identity and concentration	**E**	Line 279
Buffer/kit identity and manufacturer	**E**	Line 279
Exact chemical constitution of the buffer	D	
Additives (SYBR Green I, DMSO, etc.)	**E**	Line 279
Manufacturer of plates/tubes and catalog number	D	
Complete thermocycling parameters	**E**	Line 279
Reaction setup (manual/robotic)	D	
Manufacturer of qPCR instrument	**E**	Line 279
**qPCR VALIDATION**		
Evidence of optimisation (from gradients)	D	
Specificity (gel, sequence, melt, or digest)	**E**	Line 279
For SYBR Green I, Cq of the NTC	**E**	Line 279
Standard curves with slope and y-intercept	**E**	Line 279
PCR efficiency calculated from slope	**E**	Line 279
Confidence interval for PCR efficiency or standard error	D	
r2 of standard curve	**E**	Line 279
Linear dynamic range	**E**	Line 279
Cq variation at lower limit	**E**	Line 279
Confidence intervals throughout range	D	
Evidence for limit of detection	**E**	
If multiplex, efficiency and LOD of each assay.	**E**	
**DATA ANALYSIS**		
qPCR analysis program (source, version)	**E**	Line 281
Cq method determination	**E**	
Outlier identification and disposition	**E**	
Results of NTCs	**E**	
Justification of number and choice of reference genes	**E**	
Description of normalisation method	**E**	
Number and concordance of biological replicates	D	
Number and stage (RT or qPCR) of technical replicates	**E**	
Repeatability (intra-assay variation)	E	
Reproducibility (inter-assay variation, %CV)	D	
Power analysis	D	
Statistical methods for result significance	**E**	
Software (source, version)	E	
Cq or raw data submission using RDML	**D**	

Table 1. MIQE checklist for authors, reviewers and editors. All essential information (E) must be submitted with the manuscript. Desirable information (D) should be submitted if available. If using primers obtained from RTPrimerDB, information on qPCR target, oligonucleotides, protocols and validation is available from that source.

*: Assessing the absence of DNA using a no RT assay is essential when first extracting RNA. Once the sample has been validated as RDNA-free, inclusion of a no-RT control is desirable, but no longer essential.

**: Disclosure of the probe sequence is highly desirable and strongly encouraged. However, since not all commercial pre-designed assay vendors provide this information, it cannot be an essential requirement. Use of such assays is advised against.
